# Editorial: Electromicrobiology—from electrons to ecosystems, volume II

**DOI:** 10.3389/fmicb.2023.1253550

**Published:** 2023-07-24

**Authors:** Nils Risgaard-Petersen, Amelia-Elena Rotaru

**Affiliations:** ^1^Department of Bioscience, University of Aarhus, Aarhus, Denmark; ^2^Department of Biology, University of Southern Denmark, Odense, Denmark

**Keywords:** electromicrobiology, cable bacteria, extracellular electron transfer, bioelectrochemical technologies, *Geobacter*

## Introduction

Electromicrobiology is the study of microorganisms that have evolved the ability to exchange electrons with the extracellular environment via Extracellular Electron Transfer or EET. This ability allows them to exchange electrons with one another and to exploit electron acceptors and donors that are distantly located or cannot pass the cell envelope. As a research field, electromicrobiology is young and multidisciplinary, with many questions to address, including the diversity of EET mechanisms, cellular structures involved in EET, and the role of EET microorganisms in ecosystem functioning and future biotechnologies.

In this Research Topic “*Electromicrobiology—From Electrons to Ecosystems”—Volume II*, we collect recent advancements regarding the ecology, physiology, and applications of microorganisms capable of extracellular electron transfer. Some of the articles in this collection deal with cable bacteria, their interactions with their environment or other microorganisms, and their potential as bioremediation agents. Others deal with mechanistic aspects of extracellular electron transfer in both model and non-model organisms and biotechnologies relying on such properties.

## Diversity of EET mechanisms

To harness the metabolic potential of electroactive microorganisms, it is crucial to understand how cells exchange electrons with the extracellular environment. Until now, extracellular electron transfer (EET) has been studied primarily on model microorganisms like *Geobacter sulfurreducens* and *Shewanella oneidensis*. In *S. oneidensis*, EET has been unequivocally pinned on a porin-multiheme c-type cytochrome complex (Clarke, 2022). On the other hand, in *G. sulfurreducens*, the electron transfer mechanism is assumed to either use a network of conductive molecules (type IV pili and multiheme cytochromes/MHCs) or solely multiheme cytochromes (Clarke, 2022). Recently, Gu et al. (2021) disputed the involvement of pili in EET. Instead, the authors suggest that pili play a secretory role, helping translocate multiheme cytochromes. In this volume, Lovley presents his counterarguments pointing at the role of pili in EET. It was particularly compelling that genetic manipulations modifying the aromatic amino acid content of pili resulted in variable conductivities that correlated to variable growth performances when performing EET. For example, lessening the aromatic amino acid content leads to a 1,000-fold less conductive structure, which is ineffective for EET. On the other hand, increasing the aromatic amino acid content, leads to a 5,000-fold more conductive structure that is more effective for EET.

It remains unclear why *Geobacter* employs type IV pili alongside MHCs. They may do so to ensure access to electron acceptors that are spatially far away from the cell surface.

*Geobacter* uses different sets of multiheme c-type cytochromes (MHCs) to access electron acceptors with dissimilar reductive potentials (Clarke, 2022). To accesses electron acceptors with redox potentials below −100 mV, *Geobacter* requires CbcL on the inner membrane and OmcZ on the outer membrane. In this volume, Antunes et al. studied in detail the structure of CbcL and how it injects electrons from the inner membrane into the periplasm of the cell. CbcL formed a redox complex with a periplasmic MHC—PpcA through which electrons get injected from the inner membrane into the periplasm, from one MHC to another.

*Geobacter* can also use its MHCs to reduce toxic metal salts like Co^3+^, V^5+^, or Cr^6+^. In the present volume, Karamash et al. link the reduction of toxic metal salts like Co^3+^, V^5+^, or Cr^6+^ to Fe^2+^/heme concentrations. They observed that a mutant strain, lacking four multiheme cytochromes Δ*omcBSTE* (lacking genes for OmcB, OmcS, OmcT, OmcE), could still reduce toxic metal ions. It is likely that other extracellular multiheme cytochromes, like OmcZ, are used to reduce toxic metal ions as they were for reducing the toxic radionuclide U^6+^ (Orellana et al., 2013).

In non-model organisms, extracellular electron transfer (EET) mechanisms remain obscure. To identify essential components for EET in non-model organisms Sackett et al., used genome-wide transposon mutagenesis and high-throughput sequencing (Tn-Seq) to probe the genetic underpinnings for oxidative EET by *Thioclava electrotropha*. Only 14 genes were identified as membrane-bound and essential for oxidative EET. None of the encoded proteins contain typical redox-active centers, so how precisely *T. electrotropha* does EET remains to be uncovered.

## Cable bacteria

Cable bacteria are electrically conducting filamentous bacteria of the Desulfubulbaceae family that have been discovered 10 years ago (Pfeffer et al., 2012). Cable bacteria have been found in various environments, including marine and freshwater sediments.

One of the essential functions of cable bacteria in the environment is their role in cycling nutrients and metals (Risgaard-Petersen et al., 2012). It has been suggested that cable bacteria have a role in bioremediation (Marzocchi et al., 2020). Conflicting reports indicate that they can either have a detrimental or a beneficial effect on the environment. Detrimental effects include disturbances of nitrogen (Kessler et al., 2019; Marzocchi et al., 2022) and phosphorus cycles (Sulu-Gambari et al., 2016). Beneficial effects includes reducing methane fluxes (Scholz et al., 2020), protecting against euxinia (Seitaj et al., 2015), and against the release of contaminants (van de Velde et al., 2017). In this volume, van de Velde et al. show that cable bacteria have a seasonal impact on the flux of arsenic from uncontaminated sediments to the water column. Cable bacteria act as a barrier in spring, building an iron oxide layer that sequesters arsenic. On the other hand, during the summer, this barrier disperses when anoxia is established, and arsenic fluxes become comparable to those measured in contaminated marine sediments. Additionally, Vasquez-Cardenas et al. investigate the detoxifying role of cable bacteria on Icelandic coastal sediments exposed to fish farming where substantial sedimentation of organic-rich particle leads to euxinic conditions. Cable bacteria and other sulfur oxidizers were anticipated to act as ecosystem engineers and detoxify sulfide. However, the authors did not detect significant cable bacteria activity in these sediments. It could be because sulfide accumulated above the highest limit reported for cable bacteria activity or because it is challenging to detect the cables' electroactive signals in these fish farm sediments constantly perturbed by particle input and bioturbation. Lab tests indicate that removal of the particle input increases cable bacteria activity and that of associated microorganisms, detoxifying sulfide effectively. The authors advise removing top sediment below fish farms during the fallow periods to ensure the buildup of the cable bacteria iron curtain fighting sulfide emissions and euxinia.

The activity of cable bacteria changes the sediment geochemistry influencing the surrounding microbial communities. Cable bacteria are surrounded by a veil of microorganisms that swim around them (Thorup et al., 2021). These veil microorganisms dispersed once the cable bacterium was physically cut (Bjerg et al., 2023). In this volume, Lustermans et al. investigate whether the presence of the microbial veil is connected to a cable bacteria progression and senescence in its environment and the subsequent geochemical changes. The authors follow the progression of a *Ca*. Electronema aureum from a single cable bacterium to a cable bacteria community, the activity of its veil microbiome and local geochemical changes. They observed a veil of swarming cells surrounding living and motile cable bacteria. In contrast, dead and non-motile cable bacteria were not surrounded by their associated microbial veil. Surprisingly, geochemical parameters did not appear to impact the veil activity.

Digging deeper into the physiology of cable bacteria, Geerlings et al. investigate the dynamics of polyphosphates in cable bacteria. Polyphosphates are one of the most widely distributed biopolymers and have been observed in both Archaea and Bacteria, where they have been associated with functions like regulation of gene expression and enzyme activity, response to oxidative stress, signaling, and cation sequestration (Rao et al., 2009). For cable bacteria, polyphosphates have been observed in both marine and freshwater strains (Sulu-Gambari et al., 2016; Geerlings et al.). It has been hypothesized that these phosphates act as a substitute for ATP or protect cable bacteria against oxidative stress (Kjeldsen et al., 2019; Geerlings et al., 2020). Here, Geerlings et al. dismiss that polyphosphates are used for ATP synthesis in cable bacteria by looking at the spatial-temporal dynamics of polyphosphate using labeling experiments and state-of-the-art chemical imaging. The authors now anticipate that cable bacteria use polyphosphates to protect against oxidative stress, and perhaps for motility and gene regulation.

## Applications

Electrons are transported across the cell wall via biological polymers like pili or MHCs. These biopolymers spatially separate oxidative metabolic reactions from the reduction of the terminal electron acceptor, with the first happening inside the cell and the second outside. The properties of these biopolymers, electrical conduction, ability to self-assemble and self-repair make them attractive in fabricating sustainable and biodegradable nano-electronic devices. On the other hand, the possibility to drive spatially distant redox reactions gives rise to macroscale applications like electrochemically assisted wetland treatment.

In this volume, Bonné et al. present an insightful review regarding emerging applications for conductive biological polymers like pilins (stacked peptides rich in aromatic aminoacids), multiheme cytochromes (stacked proteins with Fe-centers) and the conductive fibers extending along cable bacteria filaments. The first, pilins, have been applied in building nanowire sensors (Smith et al., 2020). The latter, cable bacteria fibers, are centimeter–long, with conductivities above any other biological nanowires and therefore promising for biodegradable electronics (Bonné et al.).

Bioelectrochemical technologies employ electroactive microorganisms as biocatalysts. A few of the applications relying on EET-metabolisms are: promoting the degradation of organics in wastewater (Yadav et al., 2023), facilitating the bioremediation of toxic pollutants (Wang et al., 2020), sensing the presence of pollutants in the environment (Smith et al., 2020), enabling methane, or other chemical syntheses (Roy et al., 2022).

Since certain bioelectrochemical technologies are intended to bioremediate environmental pollutants from sediments, it is imperative to understand how bioelectrochemical technologies impact macrofauna in sediments. Shono et al. looked at the impact of voltage exposure on an oligochaete, *Thalassodrilides* cf. *briani*, which is often found in polluted environments and is effective at cleaning up organic pollutants (Ito et al., 2016). The oligochete responded with reversible changes in movements and metabolism in response to increased organic load and dynamic fluctuations in sediment redox potential. After heavy organic input, the sediments and the macrofauna exhibited rapid recovery. However, a too-high organic input below fish-farming rafts may be fatal to the oligochete population; therefore, monitoring the redox-potential changes could be promising for informing when fishery farms could reinitiate farming after fallow periods.

Last but not least, Peñacoba-Antona et al. present how treatment of wasteland could be bioelectrochemicaly assisted at full scale. The technology is known as METland and is expected to be an effective solution to treating wastewaters in decentralized locations. The authors monitor the bioelectrochemical behavior of two full scale METlands, one in Spain and one in Denmark. They reveal that electron current density could be used as an indicator for effective removal of organic matter.

In this volume, authors unveiled new properties of electroactive microorganisms, new physiology, and new roles in ecosystem functioning. At the nanoscale, authors showed we could develop applications for electron-conducting biopolymers. At the macroscale, the metabolic responses of electroactive microorganisms could be used to sense pollutants or applied at full scale in pollutant removal technologies like METland. Harnessing the metabolic potential of electroactive microorganisms is seen as a commodity for future sustainable technologies. Therefore, a better understanding of microorganisms capable of EET is fundamental to future developments.

## Author contributions

All authors listed have made a substantial, direct, and intellectual contribution to the work and approved it for publication.
